# Preferences for induction of labor methods in India: a qualitative study of views and experiences of women, clinicians, and researchers

**DOI:** 10.1016/j.xagr.2024.100389

**Published:** 2024-08-17

**Authors:** Lydia A. Hawker, Shuchita Mundle, Jaya P. Tripathy, Pradeep Deshmukh, Beverly Winikoff, Andrew D. Weeks, Carol Kingdon, Kate Lightly

**Affiliations:** 1 Department of Women and Children's Health, University of Liverpool, Liverpool, UK (Hawker); 2 NagpurAll India Institute of Medical Sciences, Nagpur, India (Mundle); 3 All India Institute of Medical Sciences, Nagpur, India (Tripathy and Deshmukh); 4 Gynuity Health Projects, New York, NY (Winikoff); 5 University of Central Lancashire (UCLan), Lancaster, UK (Weeks, Kingdon, Lightly)

**Keywords:** birth experiences, induction experience, labor induction, misoprostol, oxytocin, qualitative research

## Abstract

**Background:**

Induction of labor (IOL) is an increasingly common intervention, but experiences and preferences of induction methods are under-researched particularly in low -and middle-income countries. Understanding these perspectives is important to improve the childbirth experience.

**Objective:**

To explore the experiences and preferences of IOL methods for women, clinicians, and researchers in the “Misoprostol or Oxytocin for Labour Induction” (MOLI) study.

**Study Design:**

This qualitative study was based in two government hospitals in the city of Nagpur, India—one tertiary referral hospital and one women's hospital. Fifty-three semi-structured interviews with women before and after induction (between days 1 and 5 postnatal), with women recruited to the “Misoprostol or Oxytocin for Labour Induction (MOLI)” randomized controlled trial (NCT03749902). Eight focus group discussions with doctors, nurses, and trial research assistants before and during trial delivery were conducted. Thematic analysis was conducted using the Framework approach.

**Results:**

Four themes emerged: (1) *IOL methods,* (2) *impact of the study,* (3) *IOL and childbirth as one small part of the wider experiences in life*, and (4) *key moments in the childbirth experience.* For women, the safety of their baby was more important than any IOL method. Clinicians had apprehensions over misoprostol use which could affect protocol implementation; they reported that changing perception is difficult as usual practice feels “comfortable.” Women wanted to share their experiences and reported key moments during childbirth including vaginal examinations, “trying for normal,” bearing the pain, waiting, and relationships with staff.

**Conclusion:**

Women did not have a strong preference for the IOL method and viewed childbirth positively when maternal and neonatal outcomes were good. Labor pain, vaginal examinations, a normal birth, and interactions with staff impacted women's experiences.

**AJOG Global Reports at a Glance**

**Why was this study conducted?**

Induction of labor experiences and method preferences for women and clinicians in low- and middle-income countries need to be better understood.

**Key findings**

Women prioritized the safety of their baby over induction method; clinicians held a strong preference for oxytocin and expressed reluctance to change current standard practice. Women's experiences were strongly influenced by pain, length of labor, staff relationships, and vaginal examinations.

**What does this add to what is known?**

To our knowledge, this is the first alongside qualitative study in a low- and middle-income setting that comprehensively explores the views and experiences of women and clinicians. Although safety of the baby is paramount in the induction process, addressing key moments in the induction process could positively impact women's experiences.

## Introduction

Induction of labor (IOL) is a vital intervention for managing hypertensive diseases in pregnancy, where a timely birth is critical.^1^ With induction rates increasing,^2^ there is a need to enhance and prioritize the childbirth experience, not simply aiming for survival.^3^ Despite this, most IOL research has focused on metrics and quantitative outcomes; with less than 5% reporting on maternal satisfaction.^4^ There is a clear need to explore the views of women from low- and middle-income countries (LMIC), particularly women at highest risk of poorest outcome.

In India, standard practice for IOL consists of cervical ripening with prostaglandins, then amniotomy with or without oxytocin infusion.^5^ WHO and the National Institute for Health and Care Excellence recommend low-dose oral misoprostol as a suitable option for labor induction.^2^^,^^6^ Oral misoprostol offers cost-effective, heat-stable, and pump-free advantages in LMICs, with lower rates of hyperstimulation and cesarean sections compared to alternatives in a Cochrane review by Kerr at al.^7^

The qMOLI study is a qualitative sub-study of the “Misoprostol or Oxytocin for Labor Induction” (MOLI) trial conducted in Nagpur, India. The MOLI study is a randomized controlled trial comparing oral misoprostol and oxytocin for augmentation following cervical ripening with oral misoprostol (NCT040037683).^8^ qMOLI aims to evaluate women's views, experiences, priorities, and acceptability of the induction methods used, as well as clinicians’ views on the acceptability, feasibility, usability, and potential barriers to implementation of MOLI study protocols.

## Methods

The study is reported following the consolidated criteria for reporting qualitative research guidelines^9^ (Appendix Table A.1).

A qualitative design was employed alongside the MOLI study using semi-structured interviews, before and after induction, to investigate women's views and focus group discussions (FGDs) with clinicians and research staff. The focus group with research staff was conducted separately from other clinical staff and was valuable in providing a third-party perspective of both clinicians’ and women's views.

### Setting, participants, and data collection

qMOLI was run in two government hospital sites in Nagpur, central India—a tertiary referral hospital and a stand-alone women's hospital. A third MOLI site opened after qMOLI commenced, and due to similar IOL practices and patient population, it was felt this site was not sufficiently different to warrant an amendment to this study. Despite the similarities across the sites, it is unclear if women would have had the same responses. Women were eligible if they were recruited to the MOLI study and had provided informed consent. Clinicians involved in screening, recruiting, randomizing, or consenting participants to MOLI were eligible. Nurses and research assistants were also interviewed and participated in the FGDs. The full inclusion and exclusion criteria for women and clinicians can be found in the registered qMOLI protocol at ClinicalTrials.gov (NCT040037683), and the clinical results are published elsewhere.^10^

A sampling frame was outlined before recruitment to specify key characteristics such as parity, mode of birth, induction methods for women, and cadres of staff from the recruitment team. Interviews with women were conducted in January 2021 to July 2022 and continued until data saturation was reached. Interviews were conducted in the hospital setting, in the language of the patient's choice (Hindi/Marathi) by a trained research associate who was well versed in both languages, with a background in clinical research and Ayurvedic medicine. Interview schedules were devised by the research group and reviewed regularly. Two FGDs were conducted pretrial in 2019 and a further six mid-trial in September to December 2021 by a senior qualitative researcher and clinician. The COVID-19 pandemic resulted in a pause in the interviews between April to October 2020 and a delay in the mid-trial FGDs which were initially planned to be completed four to 6 months after the initial FGDs.

### Patient and public involvement

During study development, a scoping exercise was conducted with clinicians and women who had undergone induction. A local consumer representative on the MOLI Trial Steering Committee provided feedback on the protocol and interview template.

### Data analysis

This was a pragmatic study using the Framework Approach to thematic analysis.^11^^,^^12^ This approach allowed the synthesis of large volumes of data produced from interviews and FGDs and facilitated the comparison of views between different groups.

Interviews and FGDs were audio-recorded and transcribed verbatim, including observations noted. Interviews were translated into English by the interviewer and reviewed by a member of the research team. Researchers familiarized themselves with the data through postinterview meetings, note keeping, re-reading, and detailed memo writing. Codes were generated using a primarily inductive approach through open coding of the data. Two researchers (KL and CK) separately coded the first interviews and devised separate coding frameworks. Through consensus, these coding frameworks were merged. A selection of transcripts (interviews 16, 21, and focus group 1) were reviewed by the wider trial management group to develop a consensus on the coding framework, in line with the Framework Approach. Codes were clustered into categories with “other” categories to avoid missing important data.

NVivo12 software was used to code to the analytical framework before charting the data into matrices using summaries and illustrative quotes. The data was thoroughly reviewed for common patterns to generate overall themes. Any differences in the data were examined for causality and comparisons have been explored between the different groups, over multiple iterations, and group discussions.

### Reflexivity

The research team comprised of obstetricians (LH, SM, ADW, and KL), social scientists (JPT and CK), and clinical researchers with experience in maternity care and public health (PD and BW). The lack of evidence surrounding women's and clinicians’ views and experiences of misoprostol, given its suggested suitability for use in LMICs, provided the impetus for this study. We recognize that our varied backgrounds will have influence on this research and acknowledge this through reflexive accounting where possible. Regular team meetings were held throughout the study.

## Results

Fifty-three interviews were conducted with 45 women pre- or postinduction, or both (*n*=8). Women represented diverse socioeconomic and obstetric backgrounds. Postnatal interviews (days 1–5) included women receiving misoprostol (*n*=9) or oxytocin (*n*=11). Full baseline characteristics of included women can be found in Table 1. Seven focus groups with clinicians and nurses (*n*=73), and one focus group with research assistants (*n*=10), were conducted pretrial (*n*=2) or during trial delivery (*n*=6).

**Table 1:** Interview participant characteristics for pre- and postinduction interviews

Preinduction interview participant characteristics (*n*=19)		Postinduction interview participant characteristics (*n*=34)	
Characteristics	Number	Characteristics	Number
**Location**		**Location**	
GMC	7	GMC	20
Daga	12	Daga	14
**Age**		**Age**	
18–25	13	18–25	21
26–30	6	26–30	12
>30	0	>30	1
**Socioeconomic class**		**Socioeconomic class**	
Low	9	Low	14
Middle	8	Middle	11
High	0	High	1
Unknown	2	Unknown	8
**Parity**		**Parity**	
Nulliparous	14	Nulliparous	27
Multiparous	5	Multiparous	7
**Gestational age**		**Gestational age**	
<37 wk	1	<37 wk	4
37 wk+	18	37 wk+	30
		**Augmentation agent (following cervical ripening with oral misoprostol and membrane rupture)**	
		Oral misoprostol	8
		Intravenous oxytocin	11
		**Mode of birth**	
		Vaginal	14
		Cesarean	20
		**Day postnatal at interview**	
		0–1	7
		2–3	17
		4–5	10
		**Number of interviews**	
		1	26
		2	8

Four main themes were developed and are reported with illustrative quotes in Table 2: (1) IOL methods, (2) impact of the study, (3) induction and childbirth are one small part of the wider experiences in life, and (4) key moments in the childbirth experience. The full coding framework can be found in Appendix Table A.2. Common phrases and direct quotes are represented with quotation marks.

**Table 2:** Themes, subthemes, and illustrative quotes

Theme	Subtheme	Illustrative quotes
Induction of labor methods	*Previous practice and knowledge*	"[…] this is a government hospital, as long as there are chances of normal delivery, we have to try." **P4, FGD5** “I have been given the pill, that's all I know.” **P20, AN**
	*Comparison of methods*	“Misoprostol contractions, it can lead to more contractions, and we have no time for observing their contractions. Hyperstimulation leads to meconium.” **Doctor, FGD4** “When we apply Pitocin through [the] IV, they were like “got the power, got the strength.” Like this psychologically.” **Nurse, FGD3**
	*Method preference*	“[…] which medicine should be given, should be fed or should be put in saline, give me whatever you think is better. Only, there should not be any harm to my baby girl.” **P3, PN**
Impact of the study	*Feeling of importance*	“I feel you are doing very, very good work. By listening to our feelings and making improvement for others, it's a good thing. It is very good for ladies like me. They can share their feelings.” **P7, PN**
	*Counseling approach*	“They understand if we explained [to] them. If we explained [to] them with love in labour room, then they understand.” **Nurse, FGD5**
	*Implementation of protocol*	“[…] even if 3 doses are given, the findings are same […but…] the incidence of meconium-stained liquor increases. Actually, we do not go beyond third dose, then it is a failed induction” **Doctor, FGD2**
	*Difficulty changing practices*	“We are familiar with oxy [oxytocin], we are not familiar with the miso thing.” “In due days to come, the doctor will prefer […] miso-miso because you have the backbone with you” **Doctor, FGD1**
Induction and childbirth are one small part of the wider experiences in life	*Pregnancy and family*	“Everyone will care and love that baby. […] My in-laws will be very much happy” **P40, AN**
	*Centrality of the baby*	“I was trying and praying to God that my baby's life should not be in danger. On the contrary, if my life was in danger, then it was ok, but he should deliver safely.” **P43, AN** “Their importance is that the baby should come out safely, do whatever you want. They also willing to endure the pain, but [the] baby should deliver well.” **Doctor, FGD4**
	*Induction and childbirth*	“There was so much happiness […] it didn't seem like anything, like pain, happened to me” **P45, PN** "Even though, Caesar [Caesarean section] happened, whatever happened, the most important thing for me was my baby girl. I have her, safe and sound." **P5, PN**
Key moments in the childbirth experience	*Intrusive vaginal examinations*	“The worst thing for me is the vaginal examination.” **P1a, AN**
	*Pain and waiting*	“Induction of labour means rebirth, we feel as if going through death because the pain is so terrific" **P41, PN** "In some minutes, in some hours, in some days whatever it is. […] my baby will hold my hand. Then, I will see for whom I was waiting for 9 months" **P21, AN**
	*Relationships with healthcare professionals*	“They tried a lot. I respect their efforts because they tried hard for me. There is no doubt about it." **P42, PN** “I was afraid. And again, they suddenly do check-up. Don't behave properly. They assume we are like machines.” **P28, AN**

### *Women's and clinicians’ preferences*: IOL methods

Induction was seen as a “*trial*” for normal birth in government hospitals and women wanted normal birth. Cervical ripening with vaginal, sublingual, or oral misoprostol was common, followed by intravenous oxytocin after rupture of membranes.

There were, however, no departmental guidelines. Clinicians acknowledged positives and negatives of both MOLI trial regimes but preferred the familiar misoprostol/oxytocin, valuing the control in adjusting the infusion for hyperstimulation concerns. Misoprostol was felt to be “*unpredictable*” with concerns around hyperstimulation, meconium, and fetal distress. This meant that continuous monitoring was thought to be essential, although not always possible due to staff shortages or lack of equipment. The ongoing trial allayed some fears of misoprostol but there were still concerns: “*…they feel that patient may go in hyperstimulation or FHS, means fetal distress*” **(Research assistant, FGD8).** Clinicians saw logistical benefits of misoprostol as cold chain storage and proper refrigeration were unnecessary and recognized benefits for women avoiding injections and remaining mobile.

Few women had prior knowledge of induction methods but were aware of “*saline*” and “*pill*” postinduction. Clinician’ perception that women would strongly prefer oxytocin (“*saline*”) was not evident in the women's data. Women who preferred the “*pill*” felt it was a “*simple process*” **(P51, PN)** and suitable for those “*afraid of needles, injections”* **(P45, PN)**. In contrast, some women preferred oxytocin as “*saline goes into the whole veins and (pain) comes early*” **(P50, PN)**. Many women stated that they could only share what they had experienced and were not able to express their preferred method of induction or future preference if they had only experienced one method. Some women struggled to answer hypothetical questions about future choices if they were not planning another labor or induction. Other women stated they had no preference for route if both worked to bring pain and delivery; others still believed that doctors should choose the method so “*there should not be any harm*” **(P3, PN)**. Whilst women report they would discuss their induction experience with others, some were reluctant to make recommendations on method to others as it depends on the individual: “*Not everyone's body is the same*” **(P46, PN)**.

### *Women's and clinicians’ views:* Impact of the study

Study participation enabled women an opportunity to “*share their feelings*” **(P7, PN)** and gave “*importance to the patient also about what is her knowledge*” **(P46, PN)**. Clinicians evolved their views on patient counseling during the trial. Initially, some clinicians felt “*the counselling part is very much neglected… usually the patient is ill-informed*” **(Doctor, FGD1)**, whereas later expressed that if explanations were given “*with love in the labour room, they understand*” **(Nurse, FGD5)**. Mid-trial focus groups highlighted effective and compassionate counseling's role in enhancing understanding of induction indications, methods, and risks.

Concerns about misoprostol and associated meconium were echoed in pre- and mid-trial focus groups, leading to questions about the maximum number of misoprostol that should be given. Clinicians at one hospital hesitated to exceed three misoprostol doses based on prior experience: “*We do not go beyond third dose*” **(Doctor, FGD2)**. Most clinicians preferred oxytocin for augmentation as an established, “*comfortable*” regime. Clinicians did recognize that there is a reluctance to accept an “*unfamiliar*” regime and change from standard practice. One clinician suggested misoprostol might gain preference with sufficient evidence and careful patient monitoring and counseling.

### *Women's IOL experiences in context:* Induction and childbirth are one small part of the wider experiences in life

Women in this study prioritized their baby's safety over the induction method. It was “*one joyous feeling*” **(P31, PN)** to have a baby and bring family together. Family is important to women in all aspects of life but especially pregnancy where they provided advice, support, and comfort. Without social support, women could feel isolated and anxious throughout pregnancy and childbirth. The baby's well-being was central during pregnancy, induction, and childbirth, evoking both happiness and concern. This was echoed in both the women's and clinicians’ data.

Several women reported a preference for “*natural*” labor onset but appreciated the importance for the safety of both baby and mother. There is an understanding that induction is used so “*labour pain should start and there should not be any trouble for her, and she should deliver normally*” **(P42, PN)**. While most women remembered specific induction details, some found it challenging to distinguish their induction from the overall childbirth experience. For many women, the troubles of labor were forgotten when they became a mother. Women were happy with induction when their baby was safe and healthy, even with an unwanted cesarean section: “*I am happy, even though I didn't have normal*” **(P15, PN).**

### *Perceptions of interactions between women and clinician's:* Key moments in the childbirth experience

While most women viewed childbirth positively postnatally, key moments influenced some experiences. Intrusive vaginal examinations were mentioned, unprompted, in 20 of the 34 postnatal interviews as “*painful*” experiences inducing fear and embarrassment, with some women becoming tearful when recalling them. Women were prepared to “*bear pain*” in childbirth, but the experienced pain was agonizing: “*terrible labour pains came… I was screaming*” **(P16, PN)**. Once pain started, women were waiting for the pain to be over and their baby to be born. Certain women found strength to endure pain through family, staff support, or thoughts of their baby. However, some women found the pain “*unbearable*” and were unable to continue with the process: “*It was not tolerable for me… I had to do Caesar*” **(P50, PN)**. Timing and waiting were important throughout the entire hospital experience for women.

Many women reported positive interactions with staff, recalling specific individuals involved in their care. Women felt reassured when the induction and childbirth process was “*explained to me with love*” **(P40, AN)**. Having a positive relationship made women feel cared for and able to depend on staff for support and guidance. Many women appreciated doctors’ efforts to try for a normal delivery and felt respected "*that someone at least thought about me that it would be normal by having the pill*" **(P24, PN)**. However, one woman felt women were treated “*like machines*” **(P28, AN)**, experiencing abrupt checks and inadequate explanations, while two others relied on overhearing corridor conversations for care information.

## Discussion

### Principal findings

This study sought to understand women's and clinicians’ views and experiences of induction methods, alongside potential barriers to implementation of MOLI study protocols. This study reveals disparities between clinicians’ and women's priorities regarding induction methods, emphasizing the centrality of safety and effectiveness for women. Clinicians had a personal preference for oxytocin over misoprostol for augmentation, but anticipated women would share this preference due to the intravenous route. While clinicians may hesitate to adopt unfamiliar protocols like misoprostol, they acknowledge the potential for change with more evidence. The baby holds paramount importance for women throughout pregnancy and postpartum. However, the childbirth experience, including key moments influenced by clinicians, can significantly impact women's birthing experiences. This can be enhanced by fostering positive patient-staff relationships, providing ample time for counseling, and ensuring gentle and respectful maternity care, particularly during vaginal examinations.

### Results

In this study, the preferred route of induction method for women was secondary to the safety of their baby. There is limited qualitative data comparing oxytocin to oral methods of induction, particularly in LMIC settings. Women have previously expressed less satisfaction with cervical ripening methods than oxytocin,^13^ but oral misoprostol was not included in this study. However, there is evidence to suggest a preference for oral over vaginal routes.^14^^,^^15^

Clinicians shared the view that women prioritized their baby's well-being throughout induction and childbirth; this has been reported in previous studies globally.^16^^,^^17^ However, in our study, there was a strong clinician preference for oxytocin stemming from embedded concerns over misoprostol being ineffective and riskier than oxytocin.

As in this study, factors such as a lack of understanding around the induction process, length of labor, and agonizing pain have been previously reported to create a more negative experience of childbirth.^16^, ^17^, ^18^ In one study currently in preprint, there was an expectation that delivery would be by 6- and 12 hours following induction onset.^15^ Although evidence suggests induction results in a shorter duration of active labor,^19^ the time between admission and delivery can be longer,^20^ especially when considering the time taken for cervical ripening. Other studies also report that pain during induction is worse than expected, with women in a study based in Iran also using descriptors such as “unbearable” or “severe.”^21^ In the current study, there was an expectation that women should just “bear” the pain but WHO guidelines^2^ state that IOL should be conducted where analgesic options are available, and this would help provide a more positive birth experience. Vaginal examinations were associated with pain and fear for many women who reported a lack of understanding to why repeated examinations were needed, with other studies demonstrating women's wishes for fewer examinations and better counseling.^14^^,^^15^

### Clinical implications

Women in this study did want an opportunity to try for a vaginal birth with induction, if needed for the safety of their baby and themselves. In this setting, women did not prefer one route of administration despite clinicians’ expectations and preferred the safest and most effective route. Therefore, it is important that clinicians recognize personal biases and review emerging evidence with an open mind to provide the best evidence-based care.

Women have a right to respectful maternity care, with an ethical framework and bill of rights recently published by The International Federation of Gynecology and Obstetrics (FIGO).^22^^,^^23^ FIGO highlights the importance of listening and involving women and families in decision-making, providing transparent information, and delivering evidence-based practice. The induction experience could be improved by providing compassionate care with optimal counseling, analgesia, and reducing vaginal examinations.

### Research implications

This qualitative study has provided key insights into trial practices, specifically how a lack of confidence in the misoprostol/misoprostol regime could impact protocol implementation. The use of qualitative and quantitative studies together enables a more rigorous and robust interrogation, helps to understand the intricacies of clinical research, and provides early recognition of problems related to implementing new regimes.^24^

### Strengths and limitations

To our knowledge, this is the first alongside qualitative study in an LMIC setting that comprehensively explores the views and experiences of both women and clinicians, with rich data generated from a large and diverse participant base. Our study benefits from a breadth of experiences within the research team and backgrounds of the participants. Through conducting interviews and FGDs at different time points, we have been able to explore changes in perception.

This study should be considered in the context of labor induction for high-risk women in an LMIC government hospital but is in keeping with responses from other settings around painful and lengthy induction, the importance of counseling, and prioritizing baby's wellbeing.^16^, ^17^, ^18^ Therefore, the findings are felt to be relevant in the wider context, particularly with increasing induction rates globally.

The translation and transcription of interviews by a local research assistant from Marathi or Hindi to English is a recognized limitation of our study, due to the interactive role of the translator in attaching meaning and cultural clarification in cross-language qualitative research.^25^ We have tried to mitigate potential bias from the translation or interpretation of translated data through whole group discussion of the intended meaning and interpretation of data. This study was conducted partly during the COVID-19 pandemic which caused delays in data collection and some women reported an influence over hospital choice due to fears of COVID-19 patients at large government hospitals.

## Conclusion

A healthy baby is the main priority for women; they are less concerned with the induction method than being treated respectfully, understanding the induction process through considerate counseling, reducing pain and delays, and avoiding unnecessary intimate examinations. Even with pragmatic trial protocols, clinicians can be reluctant to implement regimes which are unfamiliar. Through alongside qualitative studies, we can not only explore these concerns and understand the barriers and facilitators to implementation but also gain insight into the experiences and priorities of the participants involved including how we can improve these experiences. Alongside qualitative studies should become a routine part of all clinical studies, in every setting.

## Details of ethics approval

Ethical approval was obtained in Nagpur (19/3/2019, 1756/EC/Pharmac/GMC/NGP) and the University of Liverpool (27/3/2019, 5019).

## CRediT authorship contribution statement

**Lydia A. Hawker:** Data curation, Formal analysis, Investigation, Writing – original draft. **Shuchita Mundle:** Writing – review & editing, Supervision, Resources, Project administration, Methodology, Funding acquisition, Conceptualization. **Jaya P. Tripathy:** Writing – review & editing, Methodology, Investigation, Data curation, Conceptualization. **Pradeep Deshmukh:** Writing – review & editing, Supervision, Funding acquisition, Conceptualization. **Beverly Winikoff:** Writing – review & editing, Supervision, Funding acquisition, Conceptualization. **Andrew D. Weeks:** Writing – review & editing, Supervision, Funding acquisition, Conceptualization. **Carol Kingdon:** Writing – review & editing, Supervision, Methodology, Formal analysis, Data curation, Conceptualization. **Kate Lightly:** Writing – review & editing, Supervision, Project administration, Methodology, Formal analysis, Data curation, Conceptualization.

## Appendix Supplementary materials

Image, application 1

## Footnotes

Supplementary material associated with this article can be found in the online version at doi:10.1016/j.xagr.2024.100389.
