# Supplementary material for: Preferences for induction of labor methods in India: a qualitative study of views and experiences of women, clinicians, and researchers
Source: AJOG Glob Rep. 2024 Aug 17;4(4):100389. doi: 10.1016/j.xagr.2024.100389 (PMC11415567; doi:10.1016/j.xagr.2024.100389)
Supplement: Supplementary file 1 [file mmc1.docx]

Appendix Table A.1 – COREQ

| Domain 1: Research team and reflexivity | | | Page |
| --- | --- | --- | --- |
| *a*). *Personal Characteristics* | | |  |
| 1. Interviewer/ facilitator | Which author/s conducted the interview or focus group? | A local research assistant named as contributor (RD) conducted interviews with women and author JPT conducted the focus groups with assistance from local research assistants. Another research associate PR, conducted the first pre and post interview. | 7-8 |
| 2. Credentials | What were the researcher’s credentials? *E.g*. PhD, MD | The researchers’ credentials are as follows: LH: BSc (Hons), MBChB SM: MD  JPT: MBBS, MD  PD: MD  BW: BA, MD, MPH  ADW: MB ChB DCH MD FRCOG  CK: PhD  KL: MbChB Hons  LH joined the research group following protocol formation and onset of data collection. | 1 |
| 3. Occupation | What was their occupation at the time of the study? | The researchers’ occupations are as follows: LH: Academic junior doctor  SM: Professor, Obstetrics and Gynaecology  JPT: Assistant Professor, Department of Community Medicine  PD: Professor & Head, Department of Community Medicine  BW: President, Gynuity Health Projects  ADW: Professor of International Maternal Health/Consultant obstetrician  CK: Reader in medical sociology  KL: Clinical research fellow | 9 |
| 4. Gender | Was the researcher male or female? | The researchers’ gender are as follows: LH: Female  SM: Female  JPT: Male  PD: Male  BW: Female  ADW: Male  CK: Female  KL: Female | NA |
| 5. Experience and training | What experience or training did the researcher have? | LH has previous experience in systematic reviews and completed an online course on NVIVO software and conducting qualitative research. KL was conducting her PhD at the time of the study where qualitative research has been studied in detail. SM, JPT, PD and ADW are experienced researchers with many publications and involvement in randomised controlled trials. BW and CK are expert qualitative researchers with numerous qualitative publications in various settings.  The qMOLI research team have a post-positivist stance reflecting the clinical and academic background of the team, including experts in qualitative research from India and the UK. | 9 |
| *b*). *Relationship with participants* | | | |
| 6. Relationship established | Was a relationship established prior to study commencement? | Participants were recruited from the MOLI RCT but there was no prior relationship. | 7 |
| 7. Participant knowledge of the interviewer | What did the participants know about the researcher? *e.g*. personal goals, reasons for doing the research | Participants were aware that interviews and focus groups were for research purposes, alongside the randomised controlled trial. | 7 |
| 8. Interviewer characteristics | What characteristics were reported about the interviewer/facilitator? *e.g*. Bias, assumptions, reasons and interests in the research topic | The interviewer, named as contributor (RD), is a local ayurvedic doctor with significant experience as a research assistant in many previous studies. | NA |
| Domain 2: study design | | |  |
| *a*). *Theoretical framework* | | | |
| 9. Methodological orientation and Theory | What methodological orientation was stated to underpin the study? *e.g*. grounded theory, discourse analysis etc | A pragmatic use of theory using the Framework Approach to thematic analysis. | 8 |
| *b*). *Participant selection* | | | |
| 11. Method of approach | How were participants approached? *e.g*. face-to-face, telephone, mail, email | Participants were approached in person once they had been consented to the MOLI RCT and provided with a patient information leaflet regarding the purpose of the qualitative study.  Later in the study, women with specific characteristics according to the sampling frame were approached e.g. randomised oxytocin, nulliparous, postnatal lady, with CS birth | 7 |
| 12. Sample size | How many participants were in the study? | A total of 53 interviews were conducted with 45 women, either before or after induction. 83 doctors, nurses and research assistants were included across 8 focus groups. | 9 |
| 13. Non-participation | How many people refused to participate or dropped out? Reasons? | This was a multiple step approach involving women recruited to the MOLI RCT. Some women did not wish to be included in the qualitative study but there were no women who dropped out once consented. The number of women who declined to participate was not recorded. | NA |
| *c*). *Setting* | | | |
| 14. Setting of data collection | Where was the data collected? *e.g*. home, clinic, workplace | Data were collected from women largely within a private area in the clinical area, with some interviews conducted on the ward as per participant choice. Focus groups were held in private meeting rooms within the hospitals. The study was based in two government hospitals, a tertiary referral hospital and a women’s hospital, in a large, central, urban Indian setting. | 7-8 |
| 15. Presence of non-participants | Was anyone else present besides the participants and researchers? | Most interviews were conducted with only the participant and interviewer (RD) present; however, author KL was present at the first interview and some women wished to have family present – this was clearly noted on the transcript. Focus groups were attended by participants, the interviewer JPT and local research assistants to ensure audio recording was done and consent forms filled in. KL was present for one focus group, and this was noted on the transcript. | 7-8 |
| 16. Description of sample | What are the important characteristics of the sample? *e.g*. demographic data, date | Women: age, hospital location, socioeconomic class, timing of interview (before or after induction), gestation, parity, induction method, mode of birth, number of interviews  Clinicians: hospital location, role (doctor, nurse, research assistant), timing of focus group (pre-trial or mid-trial) | 9 |
| *d*). *Data collection* | | | |
| 17. Interview guide | Were questions, prompts, guides provided by the authors? Was it pilot tested? | Interview templates were created by authors KL, CK, ADW and SM but reviewed by the full research group. The tool was piloted amongst the research team, then following the initial interviews, the interview template was reviewed. | 7-8 |
| 18. Repeat interviews | Were repeat interviews carried out? If yes, how many? | 8 women were interviewed both before and after their induction experience. | 9 |
| 19. Audio/visual recording | Did the research use audio or visual recording to collect the data? | Interviews were audio recorded. | 8 |
| 20. Field notes | Were field notes made during and/or after the interview or focus group? | Field noted were made both during and after the interviews and focus groups. | 8 |
| 21. Duration | What was the duration of the interviews or focus group? | Interview length varied between 7 minutes 59 seconds – 41 minutes 49 seconds Focus group length varied between 30 minutes 24 seconds – 70 minutes 10 seconds | NA |
| 22. Data saturation | Was data saturation discussed? | Data saturation was discussed in the methodology section.  Interview templates did involve questions around overall induction experience, mode of birth, fetal monitoring and patient reported outcome tool MGBSI, so a large number of interviews were needed to meet data saturation. Due to the sheer volume of the dataset, the research team decided to present the induction of labour data as a separate manuscript. | 7 |
| 23. Transcripts returned | Were transcripts returned to participants for comment and/or correction? | The transcripts were not returned to participants for comment or correction. This was deemed unfeasible.  As a native language speaker, the interviewer did confirm or correct understanding through discussion which added to our interpretation. | 7 |
| Domain 3: analysis and findings | | |  |
| *a*). *Data analysis* | | | |
| 24. Number of data coders | How many data coders coded the data? | Two researchers KL and CK separately coded the first interviews and devised separate coding frameworks. Through consensus, these coding frameworks were merged. A selection of transcripts (interview 16, 21 and focus group 1) were reviewed by the whole research team, including the interviewer. Emerging codes were added if necessary and presented to the research team regularly. LH and KL then coded the remaining transcripts. | 8-9 |
| 25. Description of the coding tree | Did authors provide a description of the coding tree? | Yes, primarily inductive approach through open coding of the data. | 8-9 |
| 26. Derivation of themes | Were themes identified in advance or derived from the data | The themes were derived from the data during analysis. | 8-9 |
| 27. Software | What software, if applicable, was used to manage the data? | NVivo 12 | 9 |
| 28. Participant checking | Did participants provide feedback on the findings? | Participant validation was done verbally throughout the focus groups to confirm meaning but formal member checking was not doe. Participants did not provide feedback on the findings. However, some women shared their experiences of participating in the study in the interviews, and this was a very positive experience for most women. | NA |
| *b*). *Reporting* | | | |
| 29. Quotations presented | Were participant quotations presented to illustrate the themes / findings? Was each quotation identified? *e.g*. participant number | Yes, mostly within Table 2 with some quotes in the text. Quotes from women were identified by timing of interview (prenatal or postnatal) and participant number. Quotes from focus groups were identified by role, focus group number and timing of focus group (pre-trial or mid-trial). | 10-14 |
| 30. Data and findings consistent | Was there consistency between the data presented and the findings? | Yes |  |
| 31. Clarity of major themes | Were major themes clearly presented in the findings? | Yes, major themes were clearly presented in the findings. | 10-14 |
| 32. Clarity of minor themes | Is there a description of diverse cases or discussion of minor themes? | Opposing and contradictory views are discussed with illustrative quotes provided where appropriate. Subthemes, or minor themes, are clearly displayed in table 2. | Table 2 |

Appendix Table A.2 – Coding Tree

| Code | Subtheme | Theme |
| --- | --- | --- |
| Method knowledge (women) | Previous practice and knowledge | Induction of labour methods |
| Current practice - general (clinicians) |  |  |
| Current practice - methods (clinicians) |  |  |
| Health system barriers (clinicians) |  |  |
| Miso/oxy barriers and negatives (clinicians) | Comparison of methods |  |
| Miso/oxy facilitators and positives (clinicians) |  |  |
| Miso/miso barriers and negatives (clinicians) |  |  |
| Miso/miso facilitators and positives (clinicians) |  |  |
| Staff method preference (clinicians) |  |  |
| Method preference (women) | Method preference |  |
| Future choice of method for self (women) |  |  |
| Future choice of method for others (women) |  |  |
| Family as decision makers (women) |  |  |
| Perceived women's preference (clinicians) |  |  |
| Taking part in the study (women) | Feeling of importance | Impact of the study |
| Reflecting on the interview (women) |  |  |
| IOL counselling (clinicians) | Counselling approach |  |
| Taking part in the study (clinicians) | Implementation of protocol and difficulty changing perception |  |
| Role of family (women) | Pregnancy and family | Induction and childbirth are one small part of the wider experiences in life |
| Source of knowledge (women) |  |  |
| Women's feelings (women) | Centrality of the baby |  |
| Everything was good because baby is well (women) |  |  |
| Women's worries, fears and tension (women) |  |  |
| No worries, no tension (women) |  |  |
| Women's priorities (women) |  |  |
| How to bring on labour (women) |  |  |
| Perceived women's feelings (clinicians) |  |  |
| Patient journey - antenatal (women) | Induction and childbirth |  |
| Patient journey - hospital admission (women) |  |  |
| IOL expectations (women) |  |  |
| IOL knowledge and indication (women) |  |  |
| Non-IOL preference (women) |  |  |
| Spontaneous vs IOL onset of pain (women) |  |  |
| Own previous experience of IOL (women) |  |  |
| Others' experiences of IOL (women) |  |  |
| Current experience of IOL (women) |  |  |
| Unable to recall experience (women) |  |  |
| Intrusive interventions | Intrusive vaginal examinations | Key moments in the childbirth experience |
| Pain expectation | Pain and waiting |  |
| Pain and traas |  |  |
| Timing and waiting |  |  |
| Interactions with healthcare professionals - pre-induction (women) | Relationships with healthcare professionals |  |
| Interactions with healthcare professionals - during induction (women) |  |  |
| Interactions with healthcare professionals - other |  |  |
| Trying for normal |  |  |
